# Vitamin E Microencapsulation via Electrohydrodynamic Techniques for Potential Use in Skin Care and Dermatological Applications

**DOI:** 10.3390/molecules30112306

**Published:** 2025-05-24

**Authors:** Daniela Dias, Berta Nogueiro Estevinho

**Affiliations:** 1 LEPABE—Laboratory for Process Engineering, Environment, Biotechnology and Energy, Faculty of Engineering, University of Porto, Rua Dr. Roberto Frias, 4200-465 Porto, Portugal; 2 ALiCE—Associate Laboratory in Chemical Engineering, Faculty of Engineering, University of Porto, Rua Dr. Roberto Frias, 4200-465 Porto, Portugal

**Keywords:** electrohydrodynamic techniques (EHD), electrospinning, electrospraying, vitamin E, zein, cosmetic/dermatological applications

## Abstract

Vitamin E is widely used in cosmetics and dermatological applications for its antioxidant, anti-inflammatory, and healing properties, yet its industrial use is limited by poor stability and bioavailability. To address these challenges, this study developed zein-based microstructures encapsulating vitamin E using electrohydrodynamic (EHD) techniques and evaluated how zein concentration affects morphology and release behavior. The SEM analysis showed that biopolymer (zein) concentration significantly affects microstructure morphology. At low concentrations (1%, 5%, and 15% (*w*/*v*)), micro/nanoparticles are formed, and high concentrations (30% (*w*/*v*)) yielded only fibers. The average size of the structures produced with zein (1–15% *w*/*v*) ranged from 0.38 to 0.90 µm, as measured using the program ImageJ (v1.54d). Structures containing vitamin E were generally smaller than those without. For electrospun fibers made with 30% zein, diameters ranged from 0.49 to 0.74 µm, with vitamin E-containing fibers also being thinner. Conductivity also influenced morphology; higher conductivity developed fibers, while lower conductivity formed particles. The solution with 15% (*w*/*v*) zein + 1% (*w*/*w*) vitamin E showed a conductivity of 1276 μS, similar to the 15% zein solution (1280 μS), indicating that vitamin E addition had no significant effect on conductivity. Release assays revealed that structures produced with low zein concentrations led to immediate release, while structured made with higher concentrations, prolonged release. A preliminary cosmetic formulation test has been conducted. The vitamin E microstructures were successfully incorporated into aloe vera hydrogel and coconut oil to show their potential for cosmetic applications.

## 1. Introduction

Vitamin E is a liposoluble compound essential for human health, and it is widely recognized for its antioxidant, anti-inflammatory, and skin-healing properties. Despite its benefits, its application in cosmetics and dermatology is limited by poor stability and low bioavailability, especially under industrial processing conditions [1,2]. These challenges highlight a need for innovative delivery systems that protect and enhance the performance of vitamin E in formulations. Among emerging strategies, electrohydrodynamic (EHD) techniques have shown potential for producing micro/nanostructures capable of encapsulating sensitive bioactive compounds [3,4]. However, there is limited research on the impact of polymer concentration and processing conditions on the morphology and functional performance of vitamin E-loaded structures, especially using zein as a biopolymer matrix. This study aims to address this gap by evaluating the influence of zein concentration on the morphology, size, and release behavior of vitamin E-loaded structures produced via EHD methods.

Therefore, in cosmetics, vitamin E is widely used and has shown promise in treating various dermatological conditions, including atopic dermatitis, scars, acne, wound healing, and post-pregnancy stretch marks, due to its capacity to heal by protecting fatty acids and membrane phospholipids [5,6]. Similarly, in skincare, the antioxidant activity of vitamin E makes it a valuable factor in cosmetics, protecting skin cells from UV rays and free radical damage [7,8,9,10,11], ideal for topical application. However, like other vitamins, it is highly unstable when exposed to oxygen, light, and other environmental conditions [12,13].

Microencapsulation protects vitamins by enhancing stability and effectiveness, and is widely used in food, pharmaceuticals, and cosmetics, protecting vitamins from heat, light, oxygen, and moisture, extending shelf life, improving stability and preservation, and enabling controlled, prolonged release for better bioavailability [14,15,16]. In the cosmetic industry, vitamins are microencapsulated to improve stability and enhance delivery to the skin [17]. There are numerous techniques of microencapsulation, which are further divided into chemical, physical, and physicochemical methods [16,18]. In chemical techniques, the procedures most frequently used are cross-linking, polymerization, and others. Physical microencapsulation techniques include spray-drying, freeze-drying, fluidized bed coating, extrusion, and others. Among the physicochemical techniques are supercritical fluids, simple and complex coacervation, and electrohydrodynamic techniques (EHD), such as electrospinning and electrospraying [12,19,20,21].

Electrohydrodynamic techniques are receiving attention for their advantages over other microencapsulation methods [4,22]. This technique uses a high-voltage electrostatic field to charge the polymer surface, forming microstructures with a high surface-to-volume ratio, porosity, and encapsulation efficiency [3,12,18,21]. EHD includes electrospinning, which produces micro/nanofibers, and electrospraying, which generates micro/nanoparticles. The key difference is based on the effect of the polymer concentration: higher concentrations create viscous solutions forming fibers (electrospinning), while lower concentrations produce less viscous solutions forming particles (electrospraying) [3,23,24]. Due to the complexity of these techniques, several parameters and conditions could affect the microstructures, where their modification could maximize their properties [3,19]. These factors are classified as solution parameters (viscosity, polymer concentration, molecular weight of polymer, polarity, conformation of the polymer chain, surface tension, and effect of solvents), process variables (applied voltage, spinning distance, flow rate, needle diameter, and collector distance), and environmental parameters (temperature, humidity, and air flow) [3]. In these techniques, the electrostatic repulsion of the charges on the surface of the solution and the Coulomb force of the external electric field causes the surface of the drop to be distorted, forming a cone, called a Taylor cone [4]. These forces produce a jet of charged polymer that is ejected from the tip of the Taylor cone. As the jet is driven toward the collector, dispersed distributed loads cause whipping and elongation of the jet, and rapid evaporation of the solvent occurs, causing fine micro/nanostructures to form and be deposited in the collector [24].

Considering the sensitivity of vitamin E to environmental conditions, the limitations of conventional microencapsulation methods [4,22], and the advantages of electrohydrodynamic techniques [4,25,26], these techniques have been proposed as an efficient alternative for producing microstructures with vitamin E and creating a dense and uniform barrier around the vitamin, protecting it from exposure to oxygen, light, and other environmental factors [3,21,27,28,29,30]. Furthermore, with a micro/nanoscale delivery, there is an improvement of the absorption and utilization of vitamin E in biological systems and, consequently, a controlled release of bioactive compounds [3,28,31]. Some recent research studied the microencapsulation of vitamin E through electrohydrodynamic techniques. Aytac et al. (2016) [29] encapsulated α-tocopherol (α-TOC) in β-cyclodextrin (β-CD) before electrospinning with polycaprolactone (PCL), forming an α-TOC/β-CD inclusion complex, where the resulting PCL/α-TOC/β-CD nanofiber showed enhanced antioxidant activity. Vitamin A and E were also successfully incorporated into biodegradable gelatin nanofibers through electrospinning [32], with vitamin E protecting vitamin A from oxidation, reducing degradation, and enabling prolonged release. Some other studies and patents have explored the use of electrospun nanofibers for cosmetic and pharmaceutical applications involving vitamin E. For example, Smith et al. (2021) [33] developed a facial mask incorporating vitamin E into electrospun fibers to treat skin conditions and enhance absorption. Another patent [34] presented a cosmetic sheet with controlled dissolution rates for the targeted, sustained release of vitamin E. Electrospun core–shell PAN (polyacrylonitrile) nanofibers containing magnesium l-ascorbic acid 2-phosphate (MAAP) and α-tocopherol acetate (α-TAc) demonstrated stable, sustained release and photoprotective effects [7]. Kalantary et al. (2019) [35] created PCL/gelatin nanofiber mats with vitamin E for oxidative stress protection. Other studies produced silk fibroin mats with a water-soluble form of vitamin E, promoting antioxidant activity, sustained release, and skin regeneration [36], and cellulose acetate nanofiber mats for the transdermal delivery of vitamin E and retinoic acid, achieving effective encapsulation and prolonged release [37]. Fabra et al. (2016) [25] investigated the encapsulation of α-TOC into three matrixes, namely zein, soybean protein isolate (SPI), and whey protein isolate (WPI), to develop active/bioactive bilayer films for food-packaging applications, and they discovered that zein fibers have a better encapsulation efficiency of α-TOC than the other encapsulant agents. In fact, zein, a prolamin protein comprising 35–60% of corn proteins, isolated from corn endosperm [23,38], is ideal for encapsulating compounds due to its low moisture absorption, high thermal resistance, oxygen barriers, hydrophobicity, biodegradability, and biocompatibility, and also the Food and Drug Administration (FDA) recognizes it as a safe food ingredient [38,39,40]. Other studies demonstrate that zein effectively entraps compounds, enhancing bioaccessibility and protection [41,42,43,44,45]. Coelho et al. (2021) [46] demonstrated 100% efficiency in encapsulating vitamin B12 using electrospun zein fibers, preserving vitamins during processing and storage. Based on these examples and advantages, zein was selected for this research as an encapsulating agent for EHD techniques, with potential applications in cosmetics and dermatology.

Given all the benefits of vitamin E and the advantages of EHD techniques [4], microencapsulation with these methods could enhance stability, controlled release, and skin penetration, making it ideal for cosmetic products.

Therefore, considering the limitations of vitamin E and the advantages of electrohydrodynamic techniques, this method offers a promising solution. On the other hand, Zein has been used mainly for hydrophilic molecules; its interaction and encapsulation efficiency with lipophilic compounds such as vitamin E are less explored. As a result, this research focused on developing vitamin E-loaded microstructures using zein through electrohydrodynamic processes to obtain high encapsulation efficiency and incorporating them into two cosmetic formulations for potential cosmetic and dermatological applications.

## 2. Results and Discussion

This study successfully produced biopolymeric (zein) microstructures containing vitamin E by electrospinning technology, achieving highly effective encapsulation to evaluate their possible applications in the cosmetic sector.

### 2.1. Microstructure Characterization

#### 2.1.1. Conductivity

**Figure 1:**
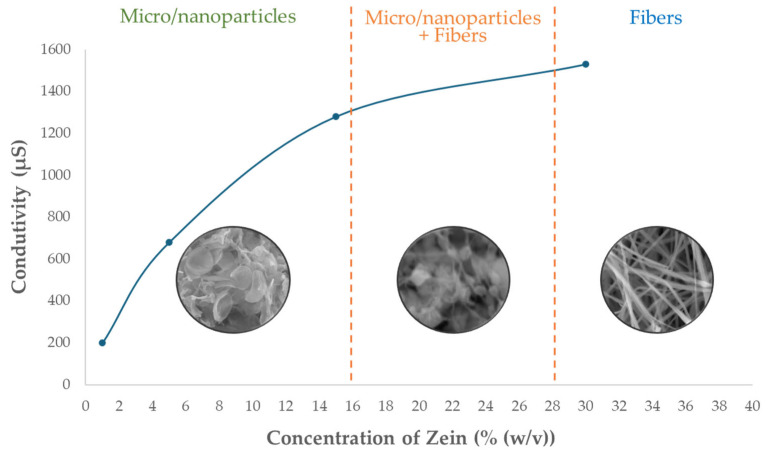
Electrical conductivity of zein solutions at different concentrations: 1%, 5%, 15%, and 30% (*w*/*v*). Measurements were performed at room temperature.

The electrical conductivity of solutions was measured before the electrospinning process, where Figure 1 represents the conductivity measured for the solutions only with zein, since this parameter can significantly influence the formation of zein fibers and the efficiency of vitamin E encapsulation [27].

It is visible in Figure 1 that the increase in the conductivity facilitates fiber formation because it leads to enhanced charge density on the surface of the electrospinning jet, facilitating greater stretching and elongation of the fibers [39,47,48]. On the other hand, low conductivity can lead to particle formation [27,47,48]. There are three different zones wherein the lowest conductivity micro/nanoparticles are produced, an intermediate zone where micro/nanoparticles plus fibers are formed, and in the highest conductivity zone, it is only fibers. The solution containing 15% (*w*/*v*) zein + 1% (*w*/*w*) vitamin E was evaluated and obtained a conductivity value equal to 1276 μS, which, compared to the 15% (*w*/*v*) zein solution in Figure 1 (1280 μS), indicates that the addition of vitamin E does not affect the conductivity of the solutions.

#### 2.1.2. pH

Evaluating the solution’s pH is essential, since it influences the effectiveness of the microstructures formed, particularly concerning vitamin E, which is extremely sensitive to severe pH conditions. Therefore, the pH was measured for all the solutions with zein, as well as the solution 15% (*w*/*v*) zein + 1% (*w*/*w*) vitamin E. All the samples demonstrated similar values of pH (pH 5), which indicates that the inclusion of vitamin E in the solutions does not change the pH of the solutions. Furthermore, pH around 5 is a suitable indicator for the application of cosmetic items that must be between pH 4 and 7 because extreme pH values should be avoided when they contain added proteins (such as vitamin E), as these substances are sensitive [49].

#### 2.1.3. Optical Microscope

**Figure 2:**
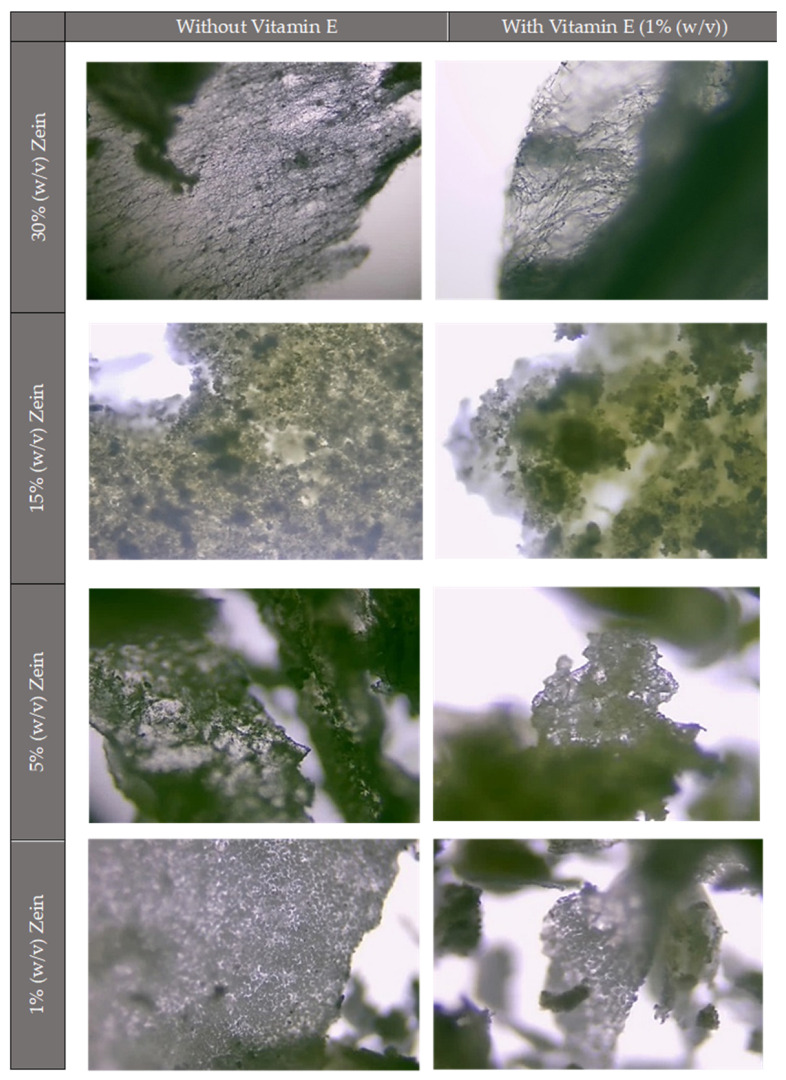
Optical micrograph of the micro/nanostructures without vitamin E and with vitamin E obtained by electrospraying/electrospinning, observed at the border region to allow light penetration through the sample. Image captured using bright-field microscopy at 10× magnification. Digital microscope with an incorporated tablet and camera (AVANTOR SN000421 2021 Microscope).

As a first method to visualize and evaluate the microstructures produced, an optical microscope was utilized. To achieve valid results, microstructures were initially created without vitamin E, allowing for comparison upon its incorporation (Figure 2).

Figure 2 shows that microstructures with higher zein concentrations (30% (*w*/*v*)) clearly show fibers and some particles, whereas the microstructures with the lowest concentrations (15%, 5%, and 1% (*w*/*v*)) only exhibit particles. Furthermore, the figure demonstrates that the microstructure’s morphology remains unchanged upon the addition of vitamin E, seeming to be nearly identical. Although these findings are in line with previous research [38], SEM images are required to more accurately describe the morphology of the microstructures since this technology offers high resolution and is more efficient at producing more detailed images. 

#### 2.1.4. Scanning Electron Microscopy (SEM)

**Figure 3:**
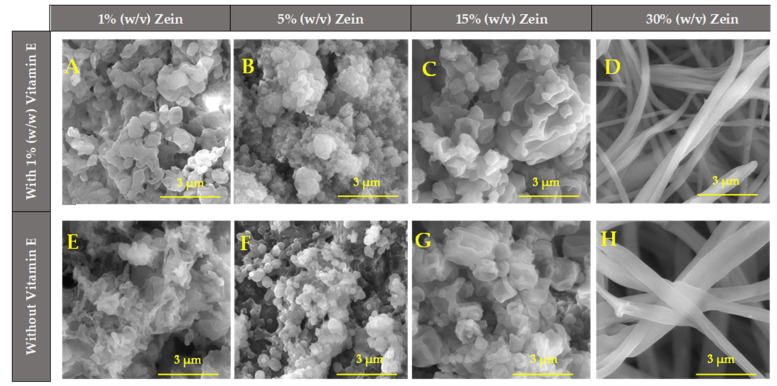
SEM images of electrospinning microstructures of different concentrations of zein with 1% (*w*/*w*) vitamin E and without vitamin E. (**A**), (**B**), (**C**), (**D**) 1% (*w*/*v*) zein + 1% (*w*/*w*) vitamin E, 5%(*w*/*v*) zein + 1% (*w*/*w*) vitamin E, 15% (*w*/*v*) zein + 1% (*w*/*w*) vitamin E, 30% (*w*/*v*) zein + 1% (*w*/*w*) vitamin E, respectively. (**E**), (**F**), (**G**), (**H**) 1% (*w*/*v*), zein, 5% (*w*/*v*) zein, 15% (*w*/*v*) zein, 30% (*w*/*v*) zein, respectively. Magnification of 30,000× for all samples, beam intensity (HV) 15.00 kV, distance between the sample and the lens (WD) less than 10.6 mm, scale bar of 3 μm for (**A**–**H**).

The morphology and size of the fibers and micro/nanoparticles were usefully characterized and confirmed (considering the optical microscope images—Figure 2) by the obtained SEM images (Figure 3). These are important factors to consider when considering industrial applications because the uniformity, similarity, and replicability of products must be guaranteed. SEM images of micro/nanostructures of zein at various concentrations (1%, 5%, 15%, and 30% (*w*/*v*)) were also generated for comparison with those containing vitamin E. The SEM images of micro/nanostructures with vitamin E (Figure 3A–D) showed no relevant differences compared to the microstructures without vitamin E (Figure 3E–H), confirming that vitamin E does not alter significantly the characteristics of the microstructures.

As previously observed in the optical microscope images, it is also possible to detect in Figure 3 that the morphology of the structures tends to change as the zein concentration rises. Higher zein concentrations (30% (*w*/*v*)) produce fibers, and smaller concentrations (1–15% (*w*/*v*)) produce particles. With intermediate concentrations between 15–30% (*w*/*v*), it is expected to obtain a mixture of fibers and particles. These results are in line with Coelho et al. (2021) [46] and Couto et al. (2023) [38], who studied the production of zein microstructures containing vitamin B12 by electrospinning and have shown similar results with identical parameters. Their studies discovered that the development of fibers is facilitated by higher zein concentrations, while the synthesis of micro/nanoparticles was confirmed at lower zein concentrations, which is consistent with our results. Figure 3A, demonstrate that the shape of the microstructures in 1% (*w*/*v*) zein + 1% (*w*/*w*) vitamin E is tiny, with flattened particles or films that are porous and have a low thickness, such as the microstructures of 1% (*w*/*v*) zein + 1% (*w*/*w*) vitamin B12 that Couto et al. (2023) [38] reported. In Figure 3B (5% (*w*/*v*) zein + 1% (*w*/*w*), vitamin E), tiny particles with rough surfaces were detected. Because of this, the technique functions as electrospray rather than electrospinning at low zein concentrations, producing fine droplets rather than fibers. As the zein concentration increases, the formation of fibers instead of microparticles should be visible. However, for the concentration of 15% (*w*/*v*) zein + 1% (*w*/*w*) vitamin E (Figure 3C), only distorted particles are still seen, which is opposite to the study carried out by Couto et al. (2023) [38], which showed that, at same concentration of 15% (*w*/*v*) zein + 1% (*w*/*w*) vitamin B12, a mixture of particles connected with fibers is visible. In this case, the process would be a hybrid operating between electrospinning and electrospray. In this case, Figure 3C shows an irregular surface of micro/nanoparticles, but it seems light starts distorting their shape, seeking to start forming fibers. This is validated for the increase in the mean size of the micro/nanostructures (Table 1. The case of 30% (*w*/*v*) zein + 1% (*w*/*w*) vitamin E, Figure 3D, is where is formed only homogeneous and continuous fibers, with a shape between tubular and ribbon-like due to the higher concentrations of solutions that are more viscous and, therefore, increase their resistance and electrical conductivity, which at these concentrations, is higher, as was seen before [50].

The mean size of the structures produced with zein (1–15% (*w*/*v*)) was evaluated with ImageJ software (v1.54d), and it was concluded that the average size ranged between 0.38 to 0.90 µm (Table 1). Therefore, micro- and nanoparticles were produced. In general, the micro/nanostructures containing vitamin E were smaller than the ones produced without it. For the case of the electrospun fibers prepared with zein 30%, the diameter of the fibers was measured (0.49–0.74 µm), and again, it was observed that the fibers containing vitamin E were thinner.

In conclusion, from the SEM images, the micro/nanostructures prepared with higher zein concentrations (30%) exhibited more compact and uniform morphologies (fibers), whereas lower concentrations resulted in more irregular structures (micro/nanostructures). This morphological difference directly influences the release behavior.

#### 2.1.5. Release Studies

Protection and controlled release are the biggest advantages of microencapsulation. The protection of the active compound is an important factor to consider, namely when it is associated with the conservation of the biological activity of the bioactive compounds. The retention of antioxidant activity in vitamin E-loaded microstructures is essential for their efficacy in dermatological applications. Additionally, encapsulation not only protects vitamin E from degradation due to light and oxygen exposure but also ensures a sustained release of the active compound, preserving its antioxidant properties over time.

Therefore, the release assays allow the evaluation of the system’s ability to release vitamin E in a controlled manner over time, since the compound release at appropriate times and under desired environments can increase the microstructures’ effectiveness according to their final application. In this study, ethanol was selected as the release medium due to its effectiveness in solubilizing vitamin E, a compound with low aqueous solubility. This choice enabled a consistent and reproducible evaluation of the release behavior from the zein-based electrospun structures. While ethanol is not physiologically representative, it serves as a useful initial model for assessing the release kinetics of hydrophobic actives. The release of the active ingredient in ethanol (70%) was total. Ethanol is commonly used in cosmetic formulations for various purposes, such as solubilizing active ingredients, enhancing absorption, and acting as a preservative. For this reason, ethanol was used as a simulated release medium to evaluate both the release profile and the stability of the micro/nanostructures in such a medium. However, it is important to note that ethanol can influence the stability of the structures. This factor is crucial for ensuring that the active ingredients are effectively delivered to the skin in a controlled manner.

**Figure 4:**
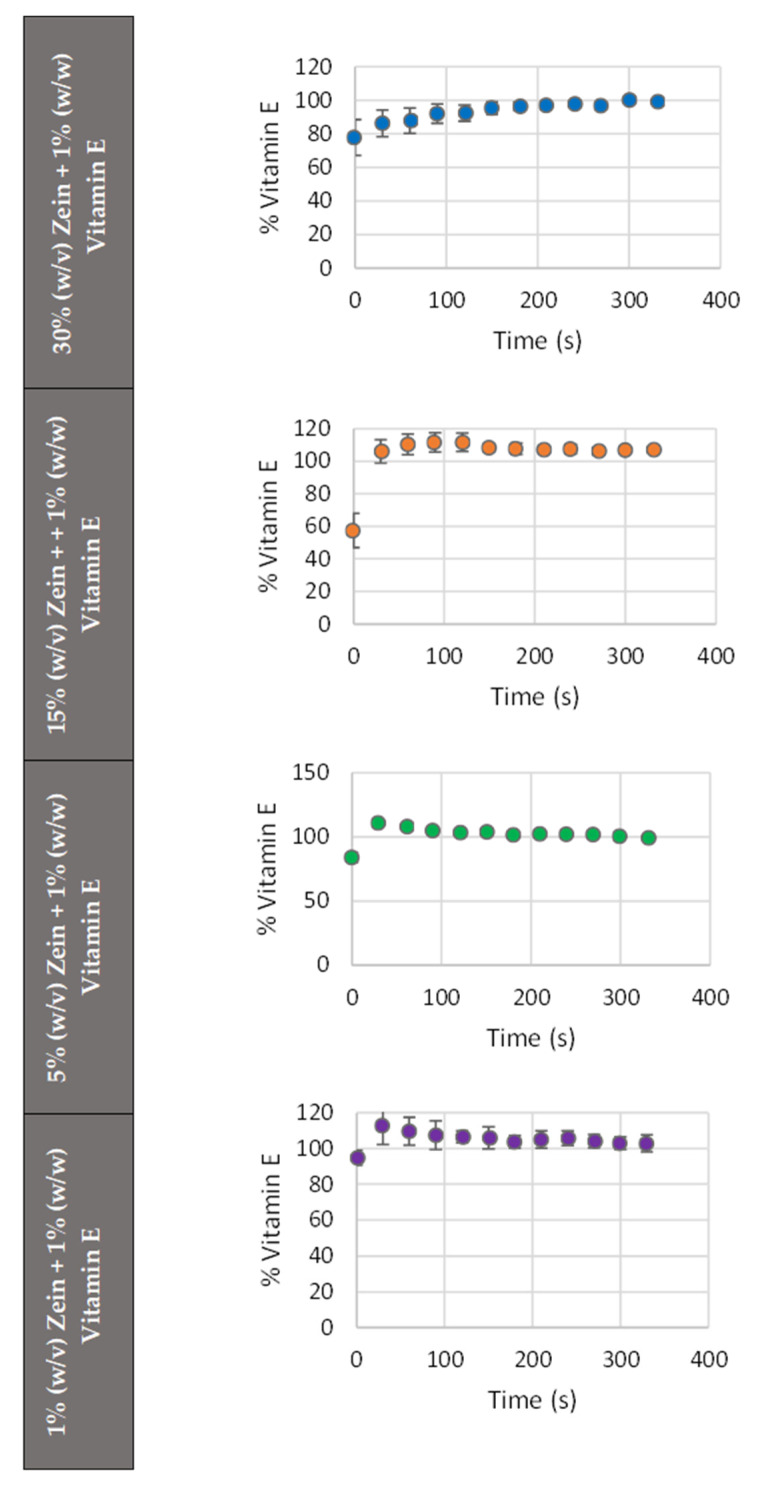
Release profiles, % of vitamin E (released) normalized by the total amount released in 70% ethanol of the microstructures loaded with 1% vitamin E in 30% (●), 15% (●), 5% (●), and 1% (●) of zein.

Figure 4 illustrates the acquired release profiles with 70% (*v*/*v*) ethanol for the samples with 1% (*w*/*w*) vitamin E and varying zein concentrations, demonstrating the percentage of release over time. Therefore, Figure 4 represents the release profiles from the microstructures loaded with 1% (*w*/*w*) vitamin E with 1% (*w*/*v*), 5% (*w*/*v*), 15% (*w*/*v*), and 30% (*w*/*v*) zein and release in 70% ethanol, in order to simulate cosmetic formulations. The micro/nanoparticles (namely 1% and 5%) have a faster initial release than the fibers (30%). This result would suggest that the amount of vitamin E released in the initial moments decreases and tends to be slower and longer as the zein concentration rises, which explains the presence of two distinct zones in the 15% and 30% release graphs: the release zone, marked by an increase of the core release, and the stabilization zone, characterized by the stability of this release. A similar fact was reported by Coelho et al. (2021) [46] with zein microstructures that were loaded with vitamin B12 and released in ethanol. When comparing the results of the vitamin E release with the ones in the article by Coelho et al. (2021) [46], in which the same encapsulating agent (Zein), the same release medium (70% ethanol), and a soluble vitamin (vitamin B12) are used, it could be verified that the release profiles are different. The distinction was that, unlike vitamin B12, the release of vitamin E did not begin at point zero, which indicates a faster initial release of vitamin E in ethanol. This faster initial release probably happens because vitamin E dissolves more quickly in ethanol than vitamin B12 because it is liposoluble [51], while B12 is water-soluble. Ethanol is a polar solvent, which facilitates the diffusion of vitamin E from the microstructures. In this case, ethanol’s high solvating power could cause a quicker disruption of the encapsulating zein matrix, leading to a faster release of the encapsulated vitamin E compared to other solvents with lower solvating capabilities.

In conclusion, lower zein concentrations (micro/nanostructures) allowed for faster diffusion, almost immediately, of vitamin E. At higher zein concentrations (30%—fibers), a more sustained release pattern is suggested. The release profiles further support this interpretation, showing a clear correlation between zein concentration and release rate. The release suggests a diffusion-controlled mechanism. In all the cases, the vitamin E release is very fast in ethanol.

The micro/nanostructures have been developed for application in the cosmetic and dermatological industries. Therefore, formulations that facilitate prolonged release times are more desirable, as some goods necessitate gradual and continuous release [13]. In these conditions, the best formulation is the 30% (*w*/*v*) zein fibers, as it facilitates extended release. These results are promising for cosmetic applications if a non-ethanolic medium is used because a prolonged release of vitamin E is desirable to maximize its antioxidant effects.

The release profiles were also useful to estimate the encapsulation efficiency, based on the methodology described in the literature [2]. This method calculates the difference between the percentage of the compound released at the end of the experiment and the amount released at time zero (representing unencapsulated material). However, due to the rapid release of vitamin E into the ethanol medium, this approach is not applicable in this case.

Based on the results obtained, it can be concluded that the microstructures formed in this study may not be suitable for use in formulations containing ethanol. The risk is that ethanol may cause the complete and premature release of the encapsulated vitamin or active ingredient before application to the skin, potentially reducing the effectiveness of the product.

### 2.2. Cosmetic Application

Vitamin E, specifically tocopherol, is a popular ingredient in skincare products due to its antioxidant, moisturizing, and skin-healing properties. Vitamin E is widely recognized as a safe and beneficial ingredient in cosmetics, with regulatory bodies like the European Union (Regulation (EC) No 1223/2009), FDA (FDA’s Cosmetic Ingredients Review (CIR)), and Health Canada (Cosmetic Regulations of the Food and Drugs Act) allowing its use in a maximum range of concentrations (typically 0.5% to 5%) for skin and hair care products. The higher concentrations of vitamin E are typically used in specific applications like anti-aging or healing products. Formulators should consider the intended use of the product, the stability of the formulation, and the skin type of the target audience when selecting an appropriate concentration of vitamin E. Vitamin E is generally well-tolerated. However, higher concentrations (above 5%) may cause irritation in sensitive individuals, particularly in leave-on products like serums or oils. It is essential to test and adjust the concentration based on the target market and product purpose.

After analyzing the vitamin E microstructures obtained through electrospinning, they were integrated into aloe vera hydrogel and coconut oil, in order to replicate a cosmetic product formulation. Aloe vera is a popular ingredient in cosmetics due to its soothing, hydrating, and anti-inflammatory properties. It is commonly found in skincare products, hair care items, and even makeup. On the other hand, coconut oil is another highly versatile and popular ingredient in cosmetics, prized for its moisturizing, anti-inflammatory, and antibacterial properties. The incorporation of vitamin E (0.1% (*w*/*w*) vitamin E/product (aloe vera/coconut oil)) resulted in distinct amounts of micro/nanostructures, depending on the zein concentrations of 5%, 15%, and 30% (*w*/*v*). Unfortunately, the incorporation of 1% zein was not feasible due to the insufficient amount that was acquired by electrohydrodynamic techniques.

#### 2.2.1. Hydrogel with Microstructures of Vitamin E

**Figure 5:**
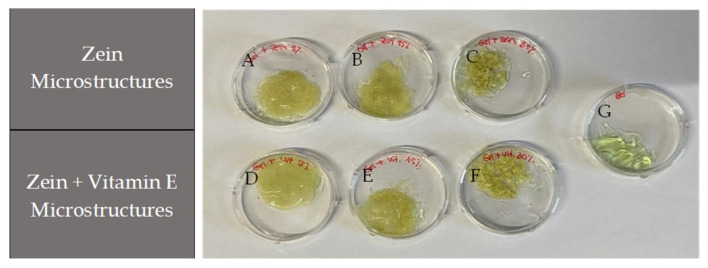
Visual incorporation of zein-based microstructures, with and without vitamin E, into aloe vera hydrogel: (**A**), (**B**), (**C**) zein microstructures 5%, 15%, 30% (*w*/*v*) (without vitamin E), respectively. (**D**), (**E**), (**F**) Zein microstructures 5%, 15%, 30% (*w*/*v*) with 1% (*w*/*v*) vitamin E, respectively. (**G**) Aloe vera hydrogel alone.

After incorporation, the combination of encapsulated vitamin E and zein with the aloe vera gel resulted in a yellowish color from the zein (Figure 5). The aloe vera gel maintained structural integrity at all concentrations without phase separation, confirming stability and demonstrating no differences between the gels without and with the microstructures.

At 5% and 15% (*w*/*v*) zein concentrations (Figure 5A and 5B, respectively), the microstructures were uniform and well-dispersed, while at 30% (fibers) (Figure 5C), aggregates appeared associated with the fiber structures. This indicates a need for adjustments in the incorporation technique or optimization of the microstructure quantity. However, the incorporation of microstructures with vitamin E (Figure 5D–F) showed better integrity than those only with zein, as shown in Figure 5A–C. These results indicate that the vitamin E microstructures produced by electrohydrodynamic techniques are compatible with aloe vera hydrogel, especially at lower concentrations, and that the integration has potential for the creation of cosmetic and dermatological formulations.

The combination of vitamin E and aloe vera gel in cosmetic formulations can be highly beneficial, as both ingredients have complementary properties that promote skin health and overall well-being. The combination of both components creates a potent skincare solution for healing, moisturizing, soothing, and protecting the skin. This combination is ideal for addressing dry skin, irritation, and sunburn and promoting overall skin health.

#### 2.2.2. Coconut Oil with Microstructures of Vitamin E

**Figure 6:**
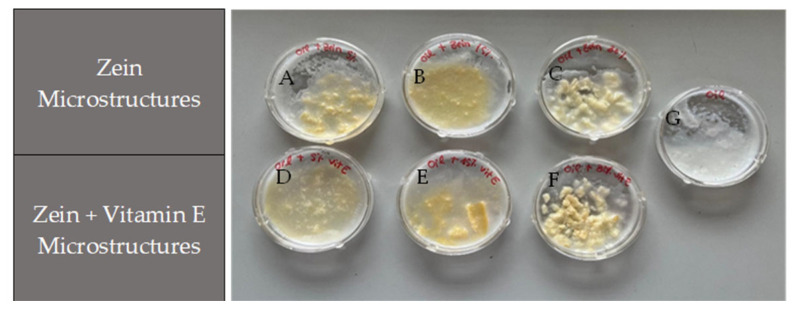
Visual incorporation of zein-based microstructures, with and without vitamin E, into coconut oil: (**A**), (**B**), (**C**) Zein microstructures 5%, 15%, 30% (*w*/*v*) (without vitamin E), respectively. (**D**), (**E**), (**F**) Zein microstructures 5%, 15%, 30% (*w*/*v*) with 1% (*w*/*v*) vitamin E, respectively. (**G**) Coconut oil.

Figure 6 represents the results of the incorporation of microstructures in coconut oil. The samples showed a yellowish color that is typical of zein, and no significant degradation or phase separation was observed.

At concentrations of 5% and 15% (*w*/*v*) of zein (Figure 6A and 6B, respectively), the microstructures were reasonably well-distributed in the oil, demonstrating acceptable incorporation capacity at lower concentrations. However, the dispersion was less homogenous in the 30% (*w*/*v*) zein samples (fibers) (Figure 6C), where the interaction between the microstructures and the oil was less efficient, with bigger visible aggregates formed, suggesting limitations in dispersibility. In contrast to what was found for the aloe vera gel, the addition of microstructures with vitamin E (Figure 6D–F) was identical to samples with the same percentage of microstructures only with zein, suggesting adjustments to the incorporation technique, necessary to improve the homogeneity at higher concentrations. Thus, coconut oil can be a viable matrix for the integration of vitamin E microstructures, especially at lower concentrations, since at high concentrations, coconut oil showed reduced capacity for the homogeneous dispersion of micro/nanostructures because it solidifies at a lower temperature, reducing its ability to make the formulation uniform. So, considering the weak incorporation capacity of the zein microstructures in coconut oil, the formulations tested could be used in exfoliant products, designed to remove dead skin cells from the surface of the skin and improve its texture and appearance. Nevertheless, coconut oil may offer additional benefits [52,53] for cosmetic and dermatological applications when combined with vitamin E, enhancing the product with moisturizing qualities. When combined, they create a powerful duo that can help improve skin health, protect from damage, and promote healing.

Therefore, the amount of micro/nanostructures that were added to formulations (oil and aloe vera gel) might be decreased and compensated for, increasing the amount of vitamin E in the micro/nanostructures as a form of optimization, as it was confirmed that there was poor dispersion of the structures in the formulations (oil and aloe vera gel) at high concentrations, in order to obtain more visually appealing results.

In conclusion, it was demonstrated that it is possible to combine the vitamin E microstructures produced by EHD techniques into two distinct types of cosmetic formulations. These formulations were found to be stable and could be employed in many types of cosmetic products, such as in the production of beauty masks containing vitamin E. However, the choice between hydrogel and oil matrix depends on the final application, such as moisturizing products (like the beauty masks) or dermatological treatments. In all the cases, the cosmetic companies must ensure that vitamin E-containing products meet safety standards, undergo adequate testing, and are properly labeled to comply with local regulations. Additionally, vitamin E used in nanoformulations (such as microparticles or nanocapsules) must undergo additional safety evaluations to ensure that the smaller particle size does not pose risks related to skin penetration or systemic exposure. This issue is subject to specific regulations in the EU, the US, and other countries.

Therefore, future research should focus on the practical application of zein-based micro/nanoparticles and nanofibers in the food, cosmetic, and biomedical fields and should explore the potential of co-encapsulation strategies [38,54,55]. Co-encapsulation offers a unique opportunity to enhance the functional performance of formulations through synergistic interactions between multiple bioactive compounds, improving their stability, bioavailability, and controlled release behavior [38,54,55]. These benefits are particularly relevant for multifunctional formulations in nutraceuticals or cosmeceuticals, where combining antioxidants, vitamins, or therapeutic agents may yield enhanced effects. However, realizing these advantages requires the development of optimized, compound-specific encapsulation systems that account for differences in solubility, stability, and interaction with the polymer matrix. Such complexity presents challenges in terms of process scalability, formulation reproducibility, and cost-effectiveness [38,54,55].

## 3. Materials and Methods

### 3.1. Reagents and Solutions

Vitamin E includes eight natural isoforms—α, β, γ, and δ-tocopherols and tocotrienols—with α-tocopherol being the most active and widely used form in food, cosmetics, and pharmaceuticals [10,30]. α-tocopherol (C29H50O2; CAS Number: 10191-41-0; molecular weight (MW): 430.71 g/mol) (Cat No. PHR1031, Lot No. LRAB3718, USA) was the bioactive compound selected to be used in this work due to its highest biological activity, and it was obtained from Sigma-Aldrich (St. Louis, MO, USA).

Zein (grade Z3625) was also purchased from Sigma-Aldrich (St. Louis, MO, USA). A 70% aqueous ethanol solution was acquired from VWR BDH-chemicals (Poole, UK) and utilized as a solvent.

### 3.2. Sample Preparation

The zein solutions were prepared by dissolving zein in an aqueous ethanol solution 70% (*v*/*v*) until completely dissolved (at room temperature, 23 °C). These ethanolic solutions were prepared with different zein concentrations (*w*/*v*, weight of zein/volume of solvent), ranging from 1% to 30% *w*/*v*. The vitamin E solutions were prepared by dissolving it in 70% (*w*/*w*) aqueous ethanol, and the concentration chosen was 1% (*w*/*w*, weight of vitamin E/weight of zein). Vitamin E solutions were added to the zein solutions, resulting in the final solutions for the EHD process. This experimental methodology was optimized based on previous works of the authors [38,46].

### 3.3. Conductivity and pH of Zein Solutions

Electrical conductivity may have a significant impact on the development of zein micro/nanostructures [27]. Before the electrospinning/electrospray process, the conductivities of the solutions (1%, 5%, 15%, and 30% (*w*/*v*) zein) were measured by a PHOENIX Instrument EC-31 multi (Garbsen, Germany). In order to verify if the addition of vitamin E changed the conductivity of the solutions, the solution with 15% (*w*/*v*) zein + 1% (*w*/*w*) vitamin E was also measured. As mentioned before, vitamin E is a compound that is very sensitive. Thus, it can be affected by pH, which can degrade in very acidic or very alkaline conditions. Additionally, because this study focuses on cosmetic applications, pH management is essential and crucial, where a slightly acidic pH (pH 4–7) is ideal for stability and compatibility with the skin. Thus, the pH of the 1%, 5%,15%, and 30% (*w*/*v*) zein solutions were measured by the device PHOENIX Instrument EC-31 multi (Garbsen, Germany), as well as the 15% (*w*/*v*) zein + 1% (*w*/*w*) vitamin E sample, to compare the corresponding pH and assess whether there was any change with the addition of vitamin E. This methodology was optimized based on previous works of the authors [38,46].

### 3.4. Production of Microstructures by EHD Techniques

The solutions formed with zein and with/without vitamin E were processed into the equipment with EHD techniques to produce micro/nanotructures (particles and fibers).

Spraybase^®^ (Dublin, Ireland) provided the electrospinning experimental setup, which includes the syringe pump where the solution is placed, which has the function of controlling the flow rate. The solution is then injected through the syringe into a metal needle that is used to electrospray the zein solutions with/without vitamin E. Then is applied a high voltage power supply (1–20 kV) that generally operates in direct current mode between the needle and the collector, and as a result, the solvent evaporates, creating fibers or particles in the collector [23,24]. Under the ideal EHD conditions determined by previous studies [38,46], the final solutions were electrosprayed with a 20 kV voltage, using a stainless-steel needle (22 gauge) at a flow rate of 2 mL/h and a distance of 5 cm between the needle and the collector. The process proceeded at room temperature (~22 °C). These parameters were selected for the following reason: applied voltage (20 kV) to ensure stable Taylor cone formation and continuous jetting without electrical discharge. The flow rate (2 mL/h) provides a steady and controlled feed of the solution, avoiding dripping or intermittent jetting. The working distance (5 cm) was selected to allow complete solvent evaporation and particle solidification before reaching the collector. The needle diameter (20 gauge) was chosen to balance precision and clogging risk, facilitating a consistent and stable jet. The room temperature (~22 °C) is necessary to ensure consistent experimental conditions. During the process, the fibers or particles are deposited in the collector and, at the end, are removed into a small plastic container. Subsequently, these samples are stored in a refrigerator at 4 °C in order to maintain their properties for future analysis.

### 3.5. Microstructure Characterization

The first microstructure analyses were carried out using a digital microscope with a tablet (AVANTOR SN000421 2021 Microscope) (Avantor, Radnor, PA, United States), and despite being a more superficial evaluation, the optical microscope was utilized in order to quickly analyze the structures and see what kind of forms they are structured in. The observation was made with dry samples and with a magnification of 4 and 10×.

Scanning electron microscopy (SEM) was performed with a Fei Quanta 400 FEG ESEM/EDAX Pegasus X4M equipment (Eindhoven, The Netherlands) at Centro de Materiais da Universidade do Porto (CEMUP), Porto, Portugal. This equipment (SEM) utilizes a powerful imaging method that uses a focused beam of electrons to scan the surface of a sample (micro or nano structures), creating high-resolution images with excellent detail, providing information about the composition and topography of the samples [56]. Prior to SEM imaging, the samples were prepared as follows:

Sample preparation: The microparticles were fixed on a brass stub using double-sided adhesive tape, ensuring that the samples were securely attached.

Coating: The samples were coated with a thin layer of gold–palladium using an SPI module sputter coater in a vacuum environment. This coating ensured proper conductivity for high-resolution imaging.

Magnification: SEM images were obtained at different magnifications (1000×, 10,000×, and 30,000×) to capture various levels of structural detail, from the overall morphology to the finer features of the microstructures.

Homogeneity: Multiple areas of each sample were examined to ensure that the particles were uniformly distributed across the surface, avoiding biased results.

Image analysis: The particle sizes and morphology were analyzed using ImageJ software (v1.54d), and six different structures were measured for each sample to ensure reliable data representation.

### 3.6. Release Assays

Release assays are important for the characterization of the system, since compound release at an appropriate time is crucial to enhance the microstructures’ effectiveness. Ethanol solutions at 70% (*w*/*w*) were employed for the in vitro release tests for all the samples, considering their applicability in cosmetic formulations. To perform the release assays, UV/Vis spectrometry (spectrometer Spec Res + (Sarspec, Portugal)) was used to measure the absorbance at a wavelength of vitamin E at 290 nm after a quantity of each sample was placed in 3 mL of ethanol, which corresponded to a concentration of 0.006 mg/mL vitamin E (estimated by mass balance), in a cuvette with magnetic stirring. Continuous absorbance readings (30 s) were used to determine the core release until the absorbance value stabilized. The encapsulation efficiency can also be estimated from the vitamin E release graphs, considering the method carried out by the authors in a previous work [2]. Three replicates of each experiment were performed at room temperature (23 °C), and a linear calibration curve was applied to assess the final concentrations of vitamin E.

### 3.7. Calibration Curve

To evaluate the implemented method and establish a relationship between the absorbance measured and the concentration of vitamin E in the samples, a linear calibration curve was created. The calibration curve of vitamin E was executed with UV/Vis spectrometry (spectrometer Spec Res + (Sarspec, Portugal)) and was prepared with nine standard solutions in ethanol, with concentrations ranging from 0.001 mg/mL to 0.15 mg/mL, with an absorbance measured of 290 nm and analysed in triplicate. In order to validate the analytical method, a set of statistical characteristics that define it must be obtained. Therefore, parameters of characterization, quantification, and repeatability of the procedure were determined (Table 2).

### 3.8. Application of Microstructures with a Cosmetic Compound

One of the cosmetic compounds utilized for the incorporation of the microstructures of vitamin E was a hydrogel with aloe vera (Cien). This compound, in gel form, is extracted from the inner part of the leaf of the aloe vera plant and is widely used in cosmetic and dermatological applications due to its numerous advantageous characteristics for the skin, such as moisturizing, anti-aging, wound healing, and anti-inflammatory benefits [57]. Taking into account these benefits and the advantages of vitamin E, the microstructures of zein and vitamin E were incorporated into the aloe vera hydrogel in order to demonstrate the applicability of these microstructures in cosmetic products and further increase their benefits. Coconut oil (SIGMA, C1758-500G) was also used for the incorporation of microstructures because this compound has gained popularity due to its beneficial properties that provide moisturizing, nourishing, and protective effects for skin and hair, and that makes it a versatile ingredient widely used in cosmetic products [52,53]. Therefore, coconut oil was chosen as a base for the incorporation of the microstructures with vitamin E to demonstrate the possible cosmetic applicability of these microstructures. For their incorporation, in this case, it was necessary to heat the coconut oil, since at room temperature, this compound is in a solid form.

According to research, vitamin E formulations in cosmetic products typically range from 0.5% to 5%, to be both safe and effective when administered [8]. To ensure safety and avoid exceeding recommended levels, a concentration of 0.1% (*w*/*w*) vitamin E was selected for the final gel and oil formulations. To synthesize 1 g of gel or oil with 0.1% of vitamin E utilizing zein microparticles containing 1% vitamin E, the ideal ratio of gel/oil and microparticles was determined to assure accuracy and effectiveness, as reported in the literature.

### 3.9. Statistical Analysis

A completely randomized design (CRD) was used to assess the influence of the zein concentration (1%, 5%, 15%, and 30% *w*/*v*) on the microstructure morphology, size, and vitamin E release behavior. Each experimental condition was prepared and analyzed in triplicate.

All the analytical determinations were performed in triplicate, and the results were expressed with standard deviations associated with the measures. The results of statistical significance were analyzed (at a level of significance of *p* ≤ 0.05) by single-factor analysis of variance (ANOVA) and Tukey’s test.

## 4. Conclusions

This study demonstrated the successful encapsulation of vitamin E into zein-based micro/nanostructures using electrohydrodynamic (EHD) techniques, providing a promising approach to enhance vitamin E’s stability and controlled release. A morphological analysis confirmed that the zein concentration strongly influences structure formation. Lower concentrations (1–15% *w*/*v*) favored micro/nanoparticles, whereas higher concentrations (30%) promoted fiber formation, especially under increased solution conductivity. Additional studies with advanced analytical techniques, such as FTIR, confocal Raman microscopy, zeta potential analysis, and laser diffraction, can help to investigate the molecular interactions, surface charge properties, and size distribution of the microstructures, providing valuable insights into stability, encapsulation mechanisms, and formulation optimization for cosmetic applications.

Release assays, under in vitro simulated conditions, showed that the encapsulation system is effective for releasing vitamin E in a controlled manner over time. At the highest concentrations, it is possible to distinguish the release and stabilization zones, while the lower concentrations have a faster initial release, so the optimal formulation is the 30% (*w*/*v*) zein fibers, where a prolonged release of vitamin E is essential to enhance its antioxidant properties.

Preliminary formulation trials with aloe vera gel and coconut oil suggested good compatibility, although formulation optimization remains necessary.

While the EHD technique demonstrated effective encapsulation, controlled release, and improved stability of vitamin E, several limitations remain. The release studies were performed under simplified in vitro conditions, and more comprehensive assessments, particularly under physiologically relevant or skin-mimicking environments, are necessary to predict in vivo behavior. Long-term stability data are also required to fully evaluate the protective effect of zein encapsulation against oxidation and degradation over time. Furthermore, although promising for small-scale applications, the scalability of EHD methods poses technical and economic challenges that must be addressed for industrial implementation. Future research should focus on optimizing encapsulation parameters for large-scale production (scale-up study and evaluation of the economic feasibility), conducting long-term stability and bioavailability studies, and evaluating the efficacy of these microstructures in finished cosmetic or dermatological formulations, which are critical parameters for industrial application in the cosmetics and dermatological sectors.

Beyond cosmetics and dermatology, the findings also suggest significant potential for application in the food industry, where controlled release and the protection of sensitive nutrients like vitamin E are equally valuable, particularly in functional foods, edible coatings, and nutraceutical delivery systems.

## Figures and Tables

**Table 1:** Average diameter of the structures prepared with different concentrations of zein (1–15% (*w*/*v*)) and diameter of the electrospun fibers * prepared with zein 30% (*w*/*v*).

Formulation	Zein (% (*w*/*v*))	Average (µm)
With 1% (*w*/*w*) Vitamin E	1	0.72 ± 0.19
	5	0.38 ± 0.21
	15	0.64 ± 0.29
	30	0.49 ± 0.17 *
Without Vitamin E	1	0.67 ± 0.35
	5	0.42 ± 0.13
	15	0.90 ± 0.69
	30	0.74 ± 0.27 *

**Table 2:** Parameters of the analytical method.

Compound	Solvent	Equation	R^2^	LOD (mg/mL)	LOQ (mg/mL)
Vitamin E	Ethanol 70%	5.72x + 0.02	0.993	0.009	0.030

## Data Availability

The original contributions presented in the study are included in the article; further inquiries can be directed to the corresponding author.

## Abbreviations

The following abbreviations are used in this manuscript:

α-TOC	α-tocopherol
α-TAc	α-tocopherol acetate
β-CD	β-cyclodextrin
EHD	Electrohydrodynamic techniques
FDA	Food and Drug Administration
MAAP	Magnesium l-ascorbic acid 2-phosphate
PAN	polyacrylonitrile
PCL	poly(ε-caprolactone)
SPI	Soybean protein isolate
WPI	Whey protein isolate
